# Exploring teams of learners becoming “WE” in the Intensive Care Unit – a focused ethnographic study

**DOI:** 10.1186/s12909-015-0414-2

**Published:** 2015-08-16

**Authors:** Helen Conte, Max Scheja, Hans Hjelmqvist, Maria Jirwe

**Affiliations:** 1 Department of Anaesthesiology and Intensive Care, Karolinska University Hospital, Huddinge, Sweden; 2 Karolinska Institutet, Department of Clinical Science Intervention and Technology, Stockholm, Sweden; 3 Department of Education, Stockholm University, Stockholm, Sweden; 4 Karolinska Institutet, Department of Neurobiology, Care Science and Society, Division of Nursing, Stockholm, Sweden

**Keywords:** Collaboration, Interprofessional, Intensive Care, Teams, Participation, Focused Ethnography

## Abstract

**Background:**

Research about collaboration within teams of learners in intensive care is sparse, as is research on how the learners in a group develop into a team. The aim of this study was to explore the collaboration in teams of learners during a rotation in an interprofessional education unit in intensive care from a sociocultural learning perspective.

**Methods:**

Focused Ethnographic methods were used to collect data following eight teams of learners in 2009 and 2010. Each team consisted of one resident, one specialist nurse student and their supervisors (n = 28). The material consisted of 100 hours of observations, interviews, and four hours of sound recordings. A qualitative analysis explored changing patterns of interplay through a constant comparative approach.

**Results:**

The learners’ collaboration progressed along a pattern of participation common to all eight groups with a chronological starting point and an end point. The progress consisted of three main steps where the learners’ groups developed into teams during a week’s training. The supervisors’ guided the progress by gradually stepping back to provide latitude for critical reflection and action.

**Conclusion:**

Our main conclusion in training teams of learners how to collaborate in the intensive care is the crucial understanding of how to guide them to act like a team, feel like a team and having the authority to act as a team.

## Background

Interprofessional Collaboration (IPC) is a process evolving through interplay where each profession participates with their expertise and negotiates an understanding vital for the teams’ function [1]. Changing levels of collaboration between intensive care professionals have been attributed to factors like education and knowledge [2], situational characteristics [3], and dimensions of authority and hierarchy in communication [2, 4, 5]. IPC requires an understanding of each other’s expertise in a team [1]. Not knowing what and when to communicate with others professions are barriers to the new graduates’ participation in IPC [6]. Collaboration improves when professionals’ self-confidence and knowledge increase through experiences of respectful interprofessional interplay [6].

The level of collaboration in intensive care teams plays a critical role and reoccurring communication failures can lead to patient harm [7]. In their specialist training in intensive care, nurses and physicians need to learn with and from each other through Interprofessional Education (IPE) [8]. Formal settings with physical proximity between professions are contexts that can trigger collaboration [9]. Interprofessional training wards have been set up in hospitals to provide training mainly for undergraduate students working together, under interprofessional supervision, for one to two week periods in patient care [10, 11]. The goals are in line with worldwide recommendations and focus on two or more professions learning how to collaborate in effective ways by working together [12]. Clinical scenarios and simulation are other means used for continually training teams in patient safety and interprofessional communication in the healthcare setting [7, 13, 14].

Learning how to collaborate can be understood as a process where learners become, and act on the presumption of being part of a particular group [15]. IPC takes place around common activities e.g. rounds in training wards where students have to explain their understanding and to each other [11]. Groups that include a heterogeneous mix of students may generate a deeper understanding by working and reflecting together in patient care [10, 16]. Research has shown that the learning patterns in teams during activities in IPE range from naive to more the complex. The more complex patterns involve interplay between action and reflection in collaborative activities [16]. Collaborative failures in student teams are often related to the fact that not all professions are involved or when activities go against the individuals’ understanding of their professional responsibilities [10, 17].

In socio-cultural learning theory the concept of guided participation refers to aspects of a learning process through which the learners’ participation in a particular group gradually becomes more legitimate by talking and acting as members of this group [18]. Exploring and finding ways how to collaborate is essential for learners developing a more legitimate participation in health care activities. Guided participation also describes the inter-personal dynamics involved in learning with others, with guidance from experienced members of a particular community of practice [19]. Supervisors at interprofessional training wards have a facilitating role guiding the team of learners to take control in their collaboration while directing them to resolve difficulties [20], creating opportunities for independent work and to break down hierarchies [21]. They need to facilitate experiential learning in small groups [22] where students need to develop a sense of ownership [23].

Research on IPC within teams of learners at an IPE unit in intensive care is sparse, as is research on how the learners in a group develop into a team. The aim of this study was to explore the collaboration in teams of learners during a rotation in an IPE unit in intensive care from a sociocultural learning perspective.

## Methods

We conducted a focused ethnographic study with an exploratory approach in 2009-2010 at the only known formal interprofessional education unit in Sweden in the intensive care (IPEICU). Focused ethnography applies ethnographic methods on a distinct issue in a smaller community [24]. Such a design differs from conventional ethnography in several crucial aspects. The field visits are shorter and the researchers usually have contextual knowledge [24, 25]. The studies are data intensive and use combinations of data collection methods in order to understand a group’s activity through observing, asking and reflecting [17]. IPC in teams of learners on a post-graduate level was explored through a socio-cultural lens where their participation in a collaborative activity was assumed to be non-mechanical and guided by the group’s rules and emerging understanding [15].

The group of researchers consisted of one Intensive Care Nurse (HC) and one Intensivist (HH) both educators with experience in supervising groups. Two external educators (MJ) and (MS) had extensive experience in learning theory and qualitative methods.

### Setting

The IPEICU was active during the day shift for one to two groups of learners per week. It was integrated within a sixteen bed high-dependency ICU with a one-to-one patient to specialist nurse ratio and a two to one intensivist and resident ratio.

The learners, residents in their specialist training and nurses in their specialist education program went through two-hour introduction session focusing on pedagogical aspects of interprofessional education. After the introduction, a group of learners (one resident and one specialist nurse student) worked together in the IPEICU for four consecutive days. Each team of learners was responsible for planning, leading and coordinating the care of one adult patient with each other and others. The medical and paramedical staff consisted of specialists, residents, auxiliary nurses, physiotherapists and specialists including surgeons, radiologists and specialists from medicine. The patients suffered from different degrees of respiratory and circulatory failure and were often sedated and intubated or in the weaning phase of their ventilation treatment.

The specialist nurse students’ work was mainly situated in close proximity to the patient whereas the residents’ work required them moving around outside the ICU. The learners’ collaborative work took place in two main areas and was supervised by a team of specialists: one specialist nurse, one specialist physician and one head supervisor (specialist nurse). The patient area contained one or four beds equipped for treating and monitoring patients. Each bed had partitions for closing off the physical space around the patients, which were consistently used by the learners in order to protect the privacy of patients during various procedures. The administrative area was the arena for the learners’ daily rounds and reflective sessions in the afternoon.

### Participants

Purposive sampling was used and all learners and supervisors scheduled in IPEICU for one term were invited to participate. One learner declined, leading to one group being excluded. Eight groups, each consisting of one specialist nurse student, one resident and two supervisors gave their informed consent and were included in the study together with the head supervisor (n = 28). Eight specialist nurse students (six women, two men), five residents (one woman, four men), eight nurse supervisors (all women), six medical supervisors (three women, three men) and one head supervisor (a man) participated.

In Sweden, after finishing their pre-graduate training to become specialists, nurses need to enroll in a one-year-long university based educational programme and physicians receive at least a five-year work based residential training programme with an accreditation set by national standard. The nurses were in the last term of their one-year specialist training and had worked as nurses between six months and twelve years before entering the program. The residents were at varying levels in their five-year specialist training in anaesthesia and intensive care. The supervisors worked in the ICU and had a minimum of 6 months of work experience as a specialist nurse or as a medical specialist. The head supervisor was a nurse with extensive experience as a specialist nurse and of interprofessional education.

### Data collection

Data collection techniques were piloted in 2009 during a two-week field visit. Due to the short nature of the learners’ rotation sound recordings of reflective sessions were added after pilot to capture reflection of action in a more structured fashion.

The data were collected during two periods of field visits (two months each) in 2009/2010 as part of a larger study. The data were collected by one of the authors (HC, an ICU nurse and educator) to ensure consistency and the data sources were triangulated; observations, interviews, reflective sessions and analytic memos to ensure trustworthiness. The eight groups’ participation in collaborative activities e.g. assessment and rounds were explored through observations. The observer (HC) took a stance of lower degree of participation and followed the groups throughout the week but did not participate in their work if not specially asked (e.g. during medical crisis). She captured both the learners and supervisors collaborative interplay by initially using pictograms and then transferring these into handwritten field notes on the same day.

Informal interviews with individuals and groups were documented in field notes that sought to capture the participants’ spontaneous reflections on their collaborative interplay and to clarify the observed participation in activities. Some of the supervisors’ and students’ reflective sessions were audio recorded and transcribed to capture reflections on the learners’ participation and supportive actions. To further explain and frame the activities, contextual data surrounding each observed team were documented and merged inductively with field notes. Personal and analytic memos were written at the end of each day capturing the researcher’s reflections on problems and possibilities in the research process and in developing theoretical ideas. These were tools to distinguish between description, meaning and theoretical content in process and merged inductively with field notes.

The material consisted of close to 100 hours of observations which included interviews, four hours of tape-recorded reflections, demographic data and analytic memos. The observations relating to the work of one of the supervisors were removed from the field notes due to uncertainty of informed consent.

### Analysis

**Fig. 1:**
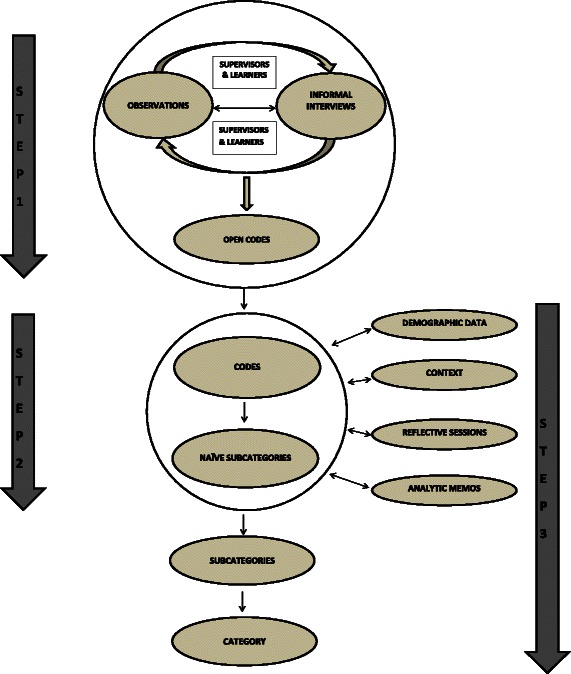
Analysis process

An iterative analysis process started parallel with the data collection and progressed through three steps (see Fig. 1) by using a constant comparative approach [26]. The first author (HC) went between the different data sources to identify and redefine emergent analytic patterns through codes and to form a theoretical description abstracting material into subcategories and category [26]. To ensure credibility of the findings the interprofessional group of authors (MS, HH & MJ) triangulated the analysis by reviewing the codes, categories and subcategories. Agreement was reached through discussion.

In the first step the data from observations and interviews in the handwritten field notes was sorted and read chronologically for each group’s activities through the week. Open codes were generated inductively representing a descriptive content of the learners and supervisors’ participation in each activity. The handwritten field notes and the open codes were turned into electronic transcripts. In the second step the materials were continually coded to identify common patterns of the progress in and between each group’s participation in collaborative activities. The learners’ and supervisors’ patterns of participation were compared chronologically. Codes were reconstructed explaining the meaning of the changing participation, the learners’ collaboration and the supervisors guiding their progress. Recurrent patterns were identified and the codes were sorted into eight naive sub-categories with descriptive headings. The final part of the analysis focused on selectively coding material on meaning of the changing participation, reviewing the consistency and variations between codes and sub-categories against demographic data of the teams, contextual factors, audio recordings from the reflective sessions and analytic memos. The naive sub-categories were first collapsed into three representing the different chronological steps in the collaboration, and then abstracted into one category representing the overall progress in collaboration. Present tense excerpts from field notes and sound recordings from the reflective sessions were used to triangulate and ground the core of the progression of the learners’ IPC in the teams.

### Ethical considerations

The regional ethical committee at Karolinska Institutet, Sweden approved the study in 2009 (Dnr 2009/5:10).

## Results

**Fig. 2:**
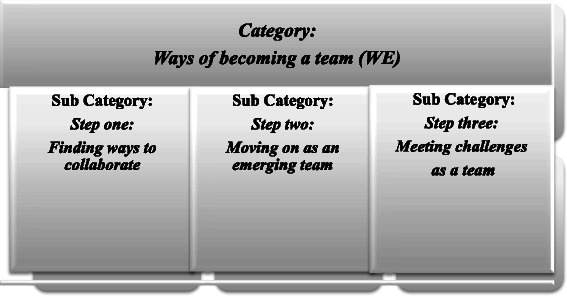
Results

The learners’ IPC progressed along a pattern of participation common to all eight groups with a chronological starting point (the first day) and an end point (the last day). The overall nature of the progress is described in the category and the distinct steps in the three subcategories (see Fig. 2).

### Ways of becoming a team (WE)

The learners’ participation in collaborative activities was described as ways leading to a goal, where the learners were guided and gradually became a team (WE). In becoming WE the learners IPC progressed in how they participated with each other and others in collaborative activities throughout the week. The learners gained substantial freedom to act together on the best course of action for the patient. As an emerging team they became increasingly autonomous in their reasoning and actively sought each other’s expertise and confirmation during collaborative activities e.g. rounds and assessments. The learners took charge and responsibility for their understanding when coordinating patient care with other professions and acted together, for instance during extubation. Their proximity to each other in activities in the IPEICU initiated and refined a collaborative interplay were they drew on, negotiated and integrated their individual and common experiences concerning the patients’ problems into a shared understanding. It was a dialectic progress and their conclusions increased in complexity by moving between exploring and comparing different professional perspectives.

The learners’ progress was paralleled by the supervisors creating opportunities and guiding the learners how to think and act together as a team. The supervisors progressively increased their space to the learners in collaborative activities. They balanced the learners’ participation in the teams’ IPC by confirming, encouraging and/or challenging them, to develop their reasoning. The learners’ were guided by active and present supervisors who used their knowledge and experiences of the demands on an ICU team’s IPC and interprofessional content. They guided the learners to expand their reasoning explaining reasons behind their interprofessional conclusions and how to plan and evaluate their actions. This manifested by, for example, what types of questions asked, when the questions were asked and who the question was directed to.

The learners’ becoming a team (WE) was a three step progress.

### Step one: finding ways to collaborate

On the first day the group of learners started to find ways to collaborate by encountering and becoming acquainted with the setting, the patient and each other. The notion of learning how to collaborate by working together in the IPEICU was not experienced as positive by all, but as challenging. Some voiced frustration over IPC being too time-consuming or wanting to focus on profession-specific skills. The groups’ patterns of interplay were, overall, cautious and four of the eight teams used avoidance strategies or engaged in outright conflicts.

*“The resident is sitting at the far end of the room with the back turned to the specialist nurse student. The supervisors are sitting at the main table waiting for the round to start. The resident is looking at the computer until the specialist physician says; it is time to start the round. Without turning around the resident answers; we don’t need a round, everything important has been decided. I just have to inform you of the plan. The special physician replies; this is not how this is done here.”* (Excerpt from field notes, rounds, team two, day one).

In starting to find each other, one of the learners – depending on who had the most experience of the patient’s health problems - took responsibility in leading their interplay throughout the activities. This commonly occurred during the group’s first round, a few hours into the day where they began to find structure in their interplay. The learners were finding ways to explore and negotiate their different understanding of the patient’s problem.

*“There is a silent moment before the resident and specialist nurse student begin the round with evaluating the goals set from the day before. The specialist physician encourages them to use the round order to structure the interplay. The resident looks at the specialist nurse student and says; I feel a little lost. Shall we also use also SBAR*^*1*^*to structure how we communicate? The specialist nurse student answers; I agree.”* (Excerpt from field notes, rounds, team three, day one).

^*1*^*A communication tool used to structure and frame conversations from the keywords:****S****ituation,****B****ackground,****A****ssessment and****R****ecommendations*

The progression manifested itself in their changing participation when the learners began to switch between statements such as “*I think”, “I observe”* (Excerpt from multiple field notes) to collaboratively focused expressions, asking for and listening to the other learners’ experience and using such expressions as *“How about you?”* “*What do you think?”* (Excerpt from multiple field notes)*.* Time wise, the focus of the groups’ interplay was on describing and then comparing what they knew from earlier theoretical and practical experiences of the patient’s problem, e.g. ventilation. The groups sought active verbal confirmation from the supervisors before moving on to negotiate and integrate different aspects in an overall interpretation of the patient’s health status, by using and combining information from multiple sources, including x-rays, findings from nursing and medical assessments for reaching a common understanding in relation to the patients’ problems. They used expressions such as *“Our understanding*” or, “*Our plan is therefore”* (Excerpt from multiple field notes) to form a plan to coordinate their common work*.* The learners actively sought verbal confirmation from their supervisors before starting up the activities, throughout each step, and before ending it. The confirmation was often permission seeking in its nature and the expertise seeking was directed from learners to supervisors both intra- and interprofessionally.

*“The resident asks the specialist nurse student open questions during the different parts of the round before moving on to the next part. Do you agree or what is your view on the patient’s social situation? The resident looks at the supervising group and asks; do you all agree?”* (Excerpt from field notes, rounds, team four, day one).

The group was guided by supervisors who used strategies focused on the learners finding ways to each other. This initially involved the supervisors stepping in and making the explicit demands on the groups’ participation in collaborative activities known. This was done both in relation to individual learners and in relation to the group as a whole before the initial patient assessment in the morning. The learners were given information about their responsibility to contribute with their experiences, primarily to use each other’s expertise in leading and coordinating work. This type of information was repeated before the round and the learners were actively redirected to seek support from each other in their interplay during rounds. The learners were guided by the supervisors to use information from written policy documents, for instance, the order of the round as a structure for collaborative activities. The supervisors expressed that the learners having proximity in collaborative activities was a crucial part in finding each other. It was in such circumstances that learners’ reflective dialogue, the negotiation of their common understanding of the patient problems and their responsibilities in care began to emerge.

*“I work with creating a physical space between myself and the learners. This is extra important since my student is cautious and quiet and it gets worse when I am too close. But I made it clear that I am here when she needs me.”* (Reflective session, supervising nurse, team two).

During the rounds the learners were guided to seek each other—both in verbal and non-verbal aspects of the interplay—by direct verbal cues and the supervisors were strategic in their proximity to the group, creating space for them. The supervisors’ participation was alternated between actively listening and actively confirming, encouraging and challenging the learners to contribute and maintaining a common structure in their interplay. Initially in common situations the learners were encouraged by their supervisors to think out loud to ensure that important information was considered, ensuring the safety and quality of care and balancing both in order to contribute with their experience in the collaborative interplay. The learners were encouraged to reach conclusions and supervisors took turns in asking comparative and open-ended questions. Whether the common conclusions were clearly understood, the planning and ways of coordinating work were often areas that were challenged.

*“The team of supervisors is leaned back in their chairs and silently observes the group’s interplay. They seek eye contact with each other and either nod or shake their head before interrupting the learners. The specialist physician starts her participation with acknowledging the complexity of the patient’s problems and then proceed to ask; so if you conclusion is that the amount of secretion is a problem - how do you plan to deal with it?”* (Excerpt from field notes, rounds, team one, day one).

At the end of day one, the reflective sessions were guided by the head supervisor focusing on the learners’ experiences and reflections on activities. Areas that needed to be improved in the group’s collaboration the following day were discussed and also how the supervisors could guide the group.

“*The first day was high pressure but the learners were in high spirits. They have established a goal oriented dialogue but we have to give them more room tomorrow so they can work on it. We also have to encourage the nurses to take command; the resident is too inexperienced and needs their support to find his role.”* (Reflective session, supervisors and head supervisor, team five and six).

The reflective session was vital for the learners’ progress the next day.

*The feedback from the reflective session the first day was powerful. It gave us a platform for how we could improve our collaboration the next day.”* (*Reflective session, specialist nurse student five)*

### Step two: moving on as an emerging team

In the morning of the second day until early afternoon on the third day, the group of learners moved on together as an emerging team and their participation progressed to attain the common and ‘WE’ as an expression used in their collaboration. Having moved through challenging situations ranging from the hesitant interplay to outright conflicts the day before, the emerging teams began to take charge in the interplay during patient assessment on the second morning. Having experience of the setting, the patient and each other, the emerging teams focused on establishing common conclusions of the changes in the patients’ health conditions and evaluating yesterday’s goals of care.

“*The resident walks in the room to left hand side of the patient’s bed up to the specialist nurse student who is finished with the morning assessment. The specialist nurse student narrates his observations and what conclusions he has drawn from his status and results of bloods samples. The resident seeks and maintains eye contact, waits quietly and then asks questions or encourages the specialist nurse student to clarity further.”* (Excerpt from field notes, morning assessment, team six, day two).

In attaining ways to question the common, the emerging teams’ participation in interplay progressed, the learners were beginning to experiment and take turns in leading their common activities. They switched smoothly between being an active listener and talker or active leader and follower through nonverbal and verbal confirmation. During the rounds the learners were actively starting up, moving through and ending parts of the common activities. Learners narrated having to negotiate their own understanding. Analyzing the other learner’s knowledge of the patient’s problem expanding what they thought they knew. Learners began to question their own need, and the needs of others, for a fuller understanding.

*“This is so complex. There are more and more dimensions to weigh in patient care and we have to do this for the patient. It is like playing a game of memory where you think you know where everything is at, but no! The board just expanded and everything is out of bounds by what the other person is saying.”* (Excerpt field notes, resident, team three).

The emergent team negotiated their conclusions and suggested actions by exploring and comparing their individual experiences from the commonly observed drawing conclusions. They connected different areas of the patients’ problems, such as decreased blood pressure, increased respiratory rate and increased heart rate. One of the learners typically said “*I believe this is sepsis, what do you think?*” (Excerpt from multiple field notes) and the other saying “*The patient might be in pain*” (Excerpt from multiple field notes). The time used on the rounds focused on questioning information, identifying their limitations and exploring where to seek that expertise. The tone of the verbal interplay was relaxed when exploring using humorous expressions or a sensitive tone while confirming the perspective of one another. The tone in the learners become critical in reaching an agreement to establish a common ground for their collaboration using cues e.g. “*So we assume”* and *“Do we agree?* (Excerpt from multiple field notes). Several of the learners challenged the other learner’s participation during collaborative activities due to persistent communication problems.

“*The resident says throughout the activity, can you please wait until I am finished, I am not done yet. The specialist nurse student goes quiet, nods and maintains eye contact.”* (Excerpt from field notes, bedside activities, team six, day three)

The learners frequently started to lead bedside activities with other professions and gave verbal directives when they needed support from their supervisors during, for instance, the bronchoscopy of a patient. The learners turned to their supervisors for verbal and non-verbal confirmation but also to sought expertise and support from each other. In reflective sessions on the first day the strengths and areas for development in the emerging teams’ interplay and the way the learners thought and acted as a team had been identified. In the second step, the learners reached and acted on collaborative focused decisions as the supervisors’ stepped back creating more space by leaving the room or directing other staff to the learners, emphasising the learners being in control in patient care. The collaborative activities, for instance, patient assessments in the morning, were observed to let the patterns of interplay be unfolded in the emergent team.

“*The supervisors are standing seven meters away from the learners who are completing their assessment. The head supervisor and specialist physician are standing with their back towards the learners and the supervising nurse alternates between observing the learners interplay and maintaining the conversation with the other supervisors.*” (Excerpt field notes, morning assessments, team eight, day two).

The learners were encouraged and challenged to attain critical conclusions by balancing uneven participation. If learners contributed unevenly they were challenged by the supervisors to answer open questions that redirected the focus of the emerging team’s discussion into comparative deductions being made. The emerging team was encouraged to make decisions based on a common understanding but to explore diversities, to maintain professional boundaries and to actively pursue and engage relevant expertise for instance from the physiotherapist. If learners consistently contributed unevenly to the work the more active learners were challenged to let the more quiet ones react, in order for new questions to be raised to be able to find common ground and directions for the IPC.

*“When the resident starts answering the question the supervising specialist nurse stops her. The supervisor redirects and repeats the question to the specialist nurse student and then everybody wait quietly for her reply.”* (Excerpt from field notes, rounds, team seven, day three).

In collaborative procedures such as the extubation of a patient, the supervisors’ guidance focused on learners establishing a step-by-step plan on how it should be done and who would do what based on professional expertise and the risk involved in the situation beforehand. The supervisors’ guidance focused on maintaining clear leadership during the procedure and afterwards reflecting on the strengths and areas for development in the collaboration. The afternoon reflective sessions were focused on evaluating the emergent teams’ experiences, views on professional responsibilities and ethical dilemmas. The supervisors’ reflective sessions focused exploring reasons behind persistent challenges in the learners IPC and finding ways how to support them.

*“There are different reasons why somebody is quiet and with the specialist nurse student we had different opinions. The specialist physician identified several situations where she believed this was due to a lack of knowledge. The specialist nurse was of the firm belief that the student was shy. I thought it was a combination of both and we agreed on limiting the amount of questions and letting them originate from the specialist nurse supervisor.*” (Reflective session, head supervisor team, seven and eight).

### Step three: meeting challenges as a team

During the final day, the third step could be seen as the team of learners reaching the endpoint of their IPC in IPEICU. The teams progressed to meet challenges and took responsibility for transferring and arguing for their common understanding of the patients’ problems while coordinating their work with other professions throughout the day. In the majority of the observed collaborative activities during the last day the team took responsibility in starting up, leading, coordinating and ending situations together e.g. in the morning assessments, and bedside care. The learners initially sought confirmation from each other, agreeing on what kind of support they needed from other experts and divided who did what and why.

*“The specialist nursing student is standing by the patient’s bed. She is monitoring vitals and talking to the patient who is not giving any response. The resident is initially sitting by a computer five meters away but is observing both the nurse and patient. The specialist student calls him over to discuss reasons the patient’s increased respiratory rate, spike in fever and unresponsiveness. They reason together and agree that the most likely cause is pneumonia but that it can also be a thrombosis. They agree that an x-ray and then blood cultures should be prioritized since the former will make a distinction between the two. The resident says I will call and coordinate with radiology.”* (Excerpt field notes, bedside, team two, day four).

In the collaborative activities, the teams’ interplay focused on what they had commonly identified as crucial aspects of the patients’ collaborative care. The teams let themselves have time to take stock of the crucial by limiting and moving on from the non-crucial aspects of care. “*Do we believe that this is relevant for the patient?*” and “*Let’s move on”* (Excerpt from multiple field notes)*.* The teams took time to explore and compare how the patient’s health status had changed over the last few days and from an interprofessional whole. They progressed on to formulate hypothetical trajectories and predictions about future outcomes for the patient. The relevance of the common explanations behind observed changes and hypothetical trajectories was negotiated and the learners verbalised their understanding together*.* The one listening in the interplay pointed out what was unclear or missing from the reasoning feeding back ideas that the team previously had discussed. The learners encouraged one another in addressing and questioning limitations in their common conclusions. It was observed in their interplay when they compared the relevance of different hypotheses explaining the patient’s problems, asked each other to give second opinions, presented alternative hypotheses and developed thoughts that could offer an explanation of the patient’s problems with expressions like: *“Do we have another explanation for this?”,* “*What are the three most logical likely reasons for this?*” and *“What speaks against it and for it?”* (Excerpt from multiple field notes).

Based on joint reflections on structure and content of their work, making connections to actions performed by the team earlier and the implications of those for the patient’s situation, learners were reaching common agreement on the direction of the work. Their joint plan as heard at the end of rounds involved the coordination of proactive care based on what the team agreed as the most likely explanations of the patient problems, and teams also reflected on how the care would impact on the health outcome and documented written care plans. The teams’ work and their verbal and non-verbal participation were actively and critically observed by the supervisors guiding the team in arguing for the relevance of the common. The clarity and relevance of the teams’ conclusions were challenged as were the teams’ use of time. Reflection on both medical and nursing aspects of the teams suggested patient care was challenged by supervisors asking the team to explicate arguments for their decisions. The team was asked questions which challenged the clarity and relevance of ideas put forward and they were asked to explain advantages and risks of suggested options and of long term consequences of treatment.

“*The supervising nurse is observing the learners interplay and her eyes increasingly move between her papers on the table and the resident. She waits until the learners have finished a part of verbal interplay and leans forward and asks; so you have both agreed on a number of things you have to do. But how should you prioritize your work and why is this relevant for the patient?”* (Excerpt field notes, rounds, team five, day four).

The reflections on the last day focused on summarizing the progress of the teams IPC and strengths and challenges in process

*“I really did not understand why I had to do this. I thought that I collaborate all the time! But now I understand that I have to have the courage to ask questions and not just accept.”*(Reflective session, Specialist nurse student three).

## Discussion

The results in the present study indicate that IPC is a process which progresses through the professionals’ participatory interplay in activities. This is confirmed by other studies [1, 2, 27] and they also suggest the necessity to explore reasons behind changes or fluctations in collaboration [2, 27]. Viewing the results in the current study through a socio-cultural lens this gradual but parallel progress in learners’ ways of participating in collaborative activities and in supervisors’ ways of supporting the learners through these activities, reveal a pattern of guided participation [18, 19, 28]. In the dynamic interface between these two movements, gaining ownership could be the explanation behind the progress. The learners become more legitimate participants and are guided by the supervisors to progress from a group towards becoming a team (WE) during their short rotation in the IPEICU. The concept of ownership has been confirmed both as central for the level of IPC in ICU teams and for training students [2, 23, 29, 30].

In the current study, becoming a team (WE) involved learners progressively acting and gaining the authority to act as team and ownership was identified as a central mechanism in other scientific studies [2, 23, 27, 29].

The three different steps of becoming a team in the current study is described as the progress of interplay between the learners which indicate that ownership is gained gradually. As an emerging team the learners become increasingly autonomous in their collaborative activities and sought each other’s support. As an emerging team their shared understanding of the patients’ problems progressed in its complexity and they increasingly coordinated their work with other professions including the necessary expertise needed for the patients care. The emerging team progressed from exploring the patient’s problems in their collaborative activities to finding a common understanding and questioning the relevance of their plans. Gaining ownership is a process where autonomy encompasses the students’ critical thinking, being aware of their limitations and seeking support in decision making [23]. In another study it was suggested that ownership requires an individual working in a team and sharing responsibility for the patient with the other members of the team [23] which is vital for an ICU team’s collaborative function [2]. A shared perception of a group’s collective ownership establishes and serves as the foundation for their identity and therefore they are able to act as a team with each other and others [2].

Of course, relevant collaborative activites [11] and finding opportunities for learners to gain ownership in their clinical work are vital [30]. Activities conflicting with their understanding of professional responsibilities lead to collaborative failures in student teams [10]. The ICU teams’ fluctuating level of collaboration have often been explained with reference to problems concerning respecting and balancing the boundaries between the individual’s in the group and their common ownership [2]. The individual ownership in a team is recognized by oneself and others through the professional’s specific contributions [2]. In the current study the learners’ progress from group towards a team was not friction free. Each of the eight teams had varied levels of experiences, and willingness to participate in collaborative activities. One or both of the learners in four teams openly expressed negative attitudes towards interprofessional education which initially created friction in the collaboration, with one person dominating or withdrawing, or in some cases led to conflicts and breakdowns. This occurred in the first of the three steps but throughout the week the teams still moved towards reasoning and acting as “WE”. Willingness, previous experiences and type of situations have been identified in other studies as important aspects influencing collaboration [1, 27, 31].

IPC evolves through interplay where professionals negotiate their understanding and act on what is vital for the patient [1]. The results in the present study suggest that the learners step forward and progressively act like a team through their collaborative activities. Participatory learning can be seen as a process through which members of a particular community of practice gains authority and move from a legitimate peripheral standpoint to a more central and active position in the group [18, 19] . Participatory learning involved active appropriation, where the learners in the IPEICU as an emerging team used cultural tools and explored, negotiated and progressively acted on their increasingly complex understanding. Describing a group of learners’ moving forward to becoming a team, it is also important to identify situations where participation reversed into earlier patterns of interplay. Analysing the teams’ collaborative activities throughout the week, in situations where the challenges for the teams became too great, the patterns of interplay could revert back to earlier steps in leadership, focus, permission and confirmation seeking. This backlash was usually short lived and in the earlier part of collaborative activities or when the supervisors actively acknowledged they, instead of the group of learners, had the ultimate responsibility for a part of patient care e.g. methods of dialysis.

Having the authority to act as a team has been suggested as a catalyst behind collaboration and is connected with who claims responsibility for action [2]. In one particular study it was confirmed that is an active process in which students are taking ownership for their part of care [23] and in another study it was suggested that the pedagogic interplay is complex but mainly driven by the supervisors [29]. In the present study, the supervisors in the IPEICU added a dimension of learners’ gaining authority to act as a team. The process where the supervisors stepped back and created space for the emerging team in the current study is clearly related to the awareness of their own authority, which was evident in the observations of the supervisors changing support. The supervisors’ reflections on actions throughout the week indicated that this was a conscious process and guiding the learners’ participation required supervisors to be present and active. A progress from the first step, where supervisors specifically stepped in and made their expectation of learners’ participation known, whereas in the second and third steps they stepped back to create space for the learners to work together on the problems.

The challenge for the supervisors in the current study was finding a balance between being supportive and providing structure on the one hand, and creating latitude for independent reflection and action, on the other. This balancing process involved encouraging or challenging individual learners while at the same time guiding the team as a whole to consolidate a firmly reflected body of common knowledge, giving the team a relevant direction for their work. Guiding the learners to move towards becoming a team also entailed focusing actions in the team, letting them establish relationships with each other and others. Initially this involved encouraging and challenging the group to find and contribute to their common work and progressively stepping back and encouraging and challenging them to question the relevance and taking charge of the common. The result also confirms how, when and why supervisors step in to intervene or step back to create space, which is vital for the teams’ gaining ownership. Supervisors stepping back too soon could lead to uncertainty or one learner taking over, while stepping forward could lead other learners to become quiet and passive or may cause conflict. This confirms what other supervisors have pointed out as crucial in supporting IPE teams: the complexity of knowing how and when to intervene [32].

When the supervisors assume the role of a guide for the team’s learning process, it gave the impression of them participating, alongside the group releasing tension in the student teams [33]. Learners express the importance of the supervisors keeping in the background, only intervening when necessary [31]. Strategies used to facilitate the students’ active participation consisted of waiting and of positive enforcement and the student driven interaction was characterized by their ownership and leading and the supervisors redirected with open questions [29]. Supervisors considered facilitating professional understanding as the true challenge in IPE [21], strategies which alternated between creating opportunities for independent work, facilitating professional understanding and breaking down hierarchies [21]. The students in one study suggested it is not enough to be located in the same place to learn together but to be supported to parctipate and connect in a contructive manner [34].

## Conclusions

Our main finding is the observation how the learners progress from groups to teams through IPC. The results describe the steps of a participatory process where the learners’ gradually gain ownership to reflect and act in collaboration. Exploring, comparing and integrating different professional perspectives led to an expanding understanding which the teams transfer while coordinating work with others. Supervisors need to be present and strategically facilitate each team’s unique need of support. The supervisors use their experiences of the demands of ICU teams’ interplay to guide the learners to find, explore, question and argue for the relevance of both individual and common contributions, and thus thinking and acting together as a team.

So to conclude, in training teams of learners how to collaborate in the ICU it is crucial to understand how to guide them becoming a team, making them feel like a team and giving them authority to act as a team.
